# Role of Maternal Dietary Peanut Exposure in Development of Food Allergy and Oral Tolerance

**DOI:** 10.1371/journal.pone.0143855

**Published:** 2015-12-10

**Authors:** Kirsi M. Järvinen, Jennifer Westfall, Magdia De Jesus, Nicholas J. Mantis, Jessica A. Carroll, Dennis W. Metzger, Hugh A. Sampson, M. Cecilia Berin

**Affiliations:** 1 Division of Allergy and Immunology, Albany Medical College, Albany, NY, United States of America; 2 Division of Infectious Diseases, Wadsworth Center, New York State Department of Health, Albany, NY, United States of America; 3 Department of Biomedical Sciences, University at Albany, Albany, NY, United States of America; 4 Center for Immunology and Microbial Diseases, Albany Medical College, Albany, NY, United States of America; 5 Division of Pediatric Allergy and Immunology & Jaffe Food Allergy Institute, The Icahn School of Medicine at Mount Sinai, New York, NY, United States of America; Cincinnati Children's Hospital Medical Center, University of Cincinnati College of Medicine, UNITED STATES

## Abstract

**Background:**

The impact of maternal ingestion of peanut during pregnancy and lactation on an offspring’s risk for peanut allergy is under debate.

**Objective:**

To investigate the influence of maternal dietary peanut exposure and breast milk on an offspring’s allergy risk.

**Methods:**

Preconceptionally peanut-exposed C3H/HeJ females were either fed or not fed peanut during pregnancy and lactation. The offsprings’ responses to peanut sensitization or oral tolerance induction by feeding antigen prior to immunization were assessed. We also assessed the impact of immune murine milk on tolerance induction pre- or post-weaning. For antigen uptake studies, mice were gavaged with fluorescent peanut in the presence or absence of immune murine milk; Peyer’s patches were harvested for immunostaining.

**Results:**

Preconceptional peanut exposure resulted in the production of varying levels of maternal antibodies in serum (and breast milk), which were transferred to the offspring. Despite this, maternal peanut exposure either preconceptionally or during pregnancy and lactation, when compared to no maternal exposure, had no impact on peanut allergy. When offspring were fed peanut directly, dose-dependent tolerance induction, unaltered by maternal feeding of peanut, was seen. Although peanut uptake into the gut-associated lymphoid tissues was enhanced by immune milk as compared to naïve milk, tolerance induction was not affected by the co-administration of immune milk either pre- or post-weaning.

**Conclusion:**

Maternal peanut exposure during pregnancy and lactation has no impact on the development of peanut allergy in the offspring. Tolerance to peanut can be induced early, even pre-weaning, by giving moderate amounts of peanut directly to the infant, and this is neither enhanced nor impaired by concurrent exposure to immune milk.

## Introduction

Peanut allergy is an example of a defect in the development of oral tolerance, which refers to the normal suppression of systemic immune responses to antigens first encountered by the oral route. The prevalence of peanut allergy has dramatically increased in children within the last decade, affecting over 1% of school-aged children in the United States, United Kingdom, Canada, and Australia. [1–4] The majority of initial reactions to peanut and tree nuts occur on the first known ingestion [5], suggesting that sensitization likely occurred through another route, such as inhalation, epicutaneous, *in utero*, or through breast milk. Because maternal, rather than paternal, atopic status has a greater effect on the atopic outcome of the progeny [6], perinatal factors including maternal diet and immune status may play a critical role in the development of peanut allergy. Although dietary antigens, such as peanut, egg, and cow’s milk proteins, have been detected in breast milk [7–10] in concentrations sufficient to cause symptoms in individuals already sensitized [10], observational studies provide mixed evidence about the potential role of exposure to peanut through breast milk in the initial sensitization phase. [11–12] All the studies possess potential recall bias and no prospective controlled human clinical trials are available. Furthermore, infant diet, which is typically linked to the maternal diet, may be a confounding factor. In fact, the recent randomized LEAP trial of oral peanut exposure in infancy showed early exposure to be highly efficacious in prevention of peanut allergy in high-risk individuals. [13] However, the role of maternal diet including or excluding peanut in the development of peanut allergy remains unclear.

A murine model of peanut allergy can be a useful tool for initial investigation of interventions for peanut allergy. [14] The advantage of using an animal study is the ability to strictly control dietary and environmental exposure. Previous animal studies on maternal exposure showed a protective effect of ovalbumin (OVA) transfer through murine milk in offspring [15–18], which was reportedly dependent on TGF-ß in milk and induction of T regulatory cells in one study [15] or vertical transfer of IgG-OVA complexes in another study. [17] We have previously shown that ante- and perinatal feeding of low dose peanut to mothers prevented sensitization and anaphylactic reactions in their offspring during the first peanut exposure. [14] Protection was associated with increased peanut-specific IgG2a, which was related to the co-administration of peanut with a mucosal adjuvant during pregnancy and lactation. While the use of an adjuvant may be useful for therapeutic purposes, our goal was to address the impact of a normal maternal dietary exposure on the risk of allergy in the offspring.

Prevention of food allergies by infant feeding practices serves as a simple, inexpensive approach to address the growing incidence of food allergy. We sought to assess the impact of maternal perinatal ingestion of peanut and other food allergens (without sensitization of mothers or co-administration with a mucosal adjuvant) on an offspring’s risk of developing an allergy or ability to develop tolerance to food. In addition, the isolated impact of breast milk on tolerance induction was assessed. Because the production of food-specific IgG antibodies is a normal physiologic phenomenon following ingestion of foods and because maternal IgG antibodies have been associated with protection against sensitization in offspring [17], we used mothers with previous exposure and presence of antibodies as biomarkers of exposure. We show that, in mice, maternal perinatal ingestion of peanut has little impact on the development of tolerance or allergy to peanut in their offspring and that the early introduction of peanut directly to the infant, in the presence or absence of breast milk, induces oral tolerance.

## Methods

### Animals, antigens and adjuvants

Six-week-old female and male C3H/HeJ mice were purchased from the Jackson Laboratory (Bar Harbor, ME). BALB/c female mice (5–8 weeks old) were obtained from Taconic Farms (Hudson, NY). Animals were maintained on peanut-free chow under conventional, specific pathogen-free conditions. At the end of the experiments, animals were sacrificed by cervical dislocation. Freshly ground whole roasted peanut for maternal feeding and oral challenges was prepared as previously described. [19] Defatted crude peanut extract (CPE) was used as an antigen in sensitization and tolerance protocols, intraperitoneal (i.p.) challenges and laboratory assays. CPE labeled with fluorescein (FITC) was used for uptake studies. [14] OVA and beta-lactoglobulin (BLG) were purchased from Sigma. Cholera toxin (CT) was purchased from List Biological Laboratories Inc (Campbell, CA).

### Ethics statement

Albany Medical College Institutional Animal Care and Use Committee (IACUC) approved the study (protocol #11–10003).

### Maternal exposure

**Fig 1:**
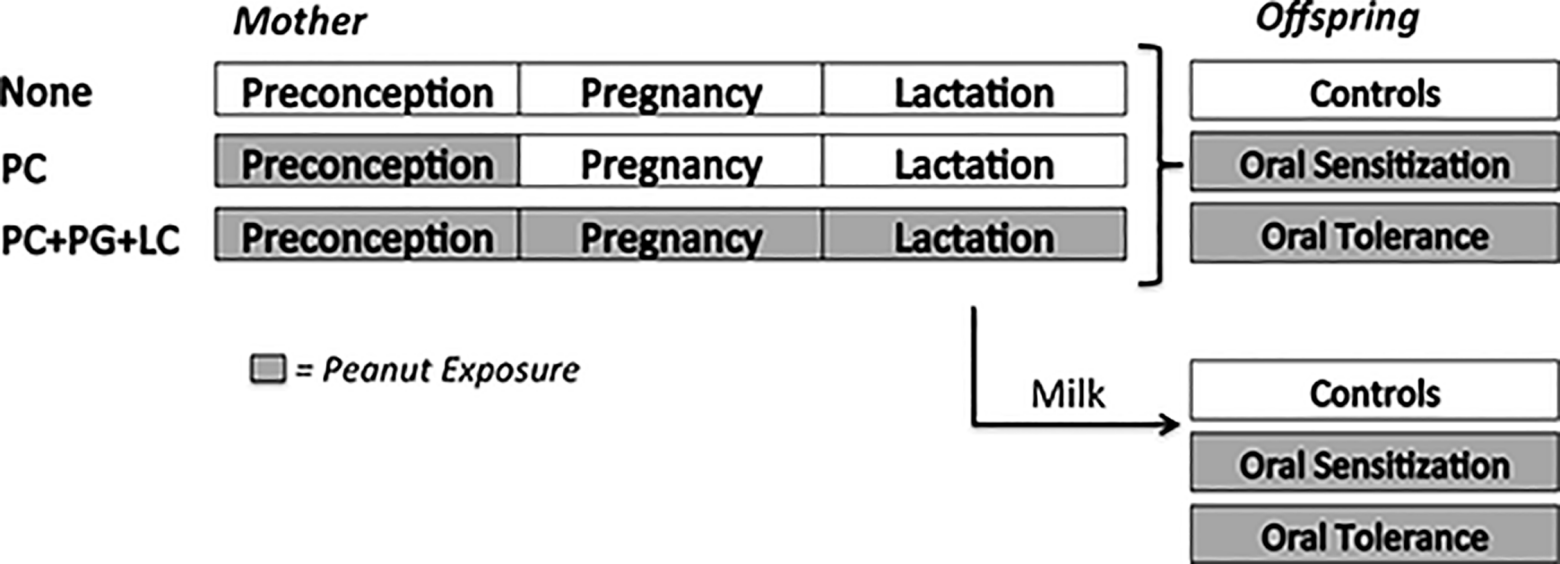
Experimental protocols. Grey shaded boxes indicate periods of oral peanut exposure. Mothers were exposed to ground peanut for 4 weeks during either in the pre-conception period only (PC), during preconception, pregnancy and lactation (PC+PG+LC); or were not fed peanut, serving as controls (None). All the offspring underwent oral sensitization to peanut by being gavage-fed crude peanut extract (CPE) with cholera toxin or were tolerized by being gavage-fed CPE without adjuvant. In some experiments, young mice born to naïve mothers were exposed to the milk of naïve (naïve milk) or peanut-exposed (immune milk) mice concurrently with CPE and cholera toxin to induce peanut sensitization or CPE alone to induce oral tolerance.

In order to determine the influence of maternal peanut ingestion during pregnancy and lactation on the development of peanut allergy in offspring, female C3H/HeJ mice were gavage-fed with ground peanut (10 mg/mouse) three times a week for four weeks prior to conception. Saphenous venous blood was collected at week 5 to determine CPE-specific IgG1, IgG2a, and IgA levels. Subsequently, they were mated with naïve males. These preconceptionally (PC) peanut-exposed mothers were divided into 2 groups for the period of pregnancy (PG) and lactation (LC): those who were gavage-fed peanut (10 mg) three times a week (PC+PG+LC) and those who were on the normal mouse chow alone, which does not contain peanut (PC). Additional peanut was left in the cages to be ingested *ad libitum* by mothers as indicated by their group assignment. Naïve mothers not fed peanut during pregnancy and lactation were used as controls (None). These mothers were then used as breeders for mice utilized in the following experiments of sensitization or tolerance induction as described below; alternatively only their milk was used, as shown in **Fig 1**. (Please see **S1A Fig** for a detailed protocol on maternal feeding.) These mothers develop IgG, but not IgE antibodies to peanut, and are not clinically reactive to peanut. Offspring of these mothers were weaned between 3.5 and 4 weeks of age. Additional groups included preconceptionally peanut exposed mothers who were fed peanut only during pregnancy and those who were fed peanut only during lactation (not shown).

### Sensitization, allergen challenge and assessment of anaphylaxis

Five-week old C3H/HeJ offspring were sensitized with CPE+CT by gavage (5 mg CPE + 20 μg CT/mouse) at weekly intervals for 6 weeks followed by 2 boosters at 2-week intervals (50 mg CPE + 20 μg CT/mouse), as previously published [20]. (Please see **S1B Fig** for a detailed protocol on offspring sensitization.) Offspring were bled prior to sensitization at week 5 and post-sensitization at week 15 of life, and peanut-specific IgE, IgG1, IgG2a, and IgA were measured by ELISA, as described below. Two weeks after the last booster, an oral challenge with 100 mg peanut i.g. was performed, followed by an intraperitoneal injection of 100 μg CPE after 30 minutes. Anaphylactic signs were evaluated 30 minutes after the challenge dose, according to a previously published scoring system. [19] Rectal temperatures were measured 30 minutes after the peanut challenge with a thermal probe (WPI Instrument, Sarasota, FL). Mast cell protease-1 (MMCP) was measured within 1 hour post-challenge utilizing an ELISA kit (R&D Systems). Splenocytes were harvested for cultures as described below.

### Tolerance induction

To determine the influence of maternal peanut ingestion on the development of oral tolerance in offspring, we performed a classic oral tolerance protocol on offspring. Offspring were weaned between 3.5 and 4 weeks of age, bled, and immediately gavage-fed 1 mg CPE daily for 5 days, followed by two immunizations with 0.1 mg CPE with alum i.p. after 2 weeks at weekly intervals. (Please see **S1C Fig** for a detailed protocol on tolerance induction.) Offspring that were not fed CPE (fed bicarbonate alone) prior to immunization served as controls. Peanut-specific IgE, IgG1, IgG2a, and IgA were measured by ELISA, as described below. This model is not sufficiently sensitized to assess symptomatic anaphylaxis.

### Impact of breast milk on tolerance induction

The role of breast milk on tolerance induction was assessed in 11-12-day-old and 3–4 week-old mice (born to naïve mothers) that were subjected to a classic oral tolerance protocol. (Please see **S1D Fig** for a detailed protocol on the impact of breast milk.)

These offspring were fed 1 mg CPE i.g. daily for 5 days, followed by 2 immunizations with CPE in alum after 2 weeks at weekly intervals. Alternatively, CPE was administered in the presence of naïve or immune milk (i.e. milk from peanut-exposed animals). Offspring that were not fed CPE (fed bicarbonate alone) prior to immunization served as controls. Offspring were bled a week after the last immunization.

In addition, the impact of breast milk on sensitization was assessed in 3–4 week-old mice that were exposed to OVA in the presence or absence of immune or naïve milk, followed by OVA epicutaneous sensitization [21] and challenge as described in Supplemental Methods.

### Cell culture and cytokine measurements

Splenocytes derived from sensitized offspring were isolated using standard techniques. After the red blood cells were lysed, splenocytes were resuspended in RPMI 1640, supplemented with fetal bovine serum. Cells were cultured in 24-well plates (4×10^6^/well/mL) in the presence or absence of CPE (200 μg/mL). Supernatants were collected after 72 hours and IL-5, IL-10, IL-13, and IFN-γ levels were determined by ELISA, according to the manufacturer’s instructions (EBioscience Affymetrix Inc, San Diego, CA).

### Measurement of antibodies

Breast milk was collected using an electric murine breast pump and processed. [22] Serum and milk samples were stored at -80°C until analyzed. CPE-specific IgE in serum was measured by a capture enzyme-linked immunosorbent assay. [23] CPE-specific IgG1, IgG2a, and IgA were measured by applying serum and breast milk dilutions to CPE-coated plates and detected with biotinylated anti-IgG1, IgG2a, and IgA, respectively, and total IgE was measured using an ELISA kit (BD Pharmingen, San Diego, CA).

### *In vivo* antigen uptake studies

BALB/c mice have been used in our laboratories in the past to assess antigen uptake. They were gavaged with 0.5 mg/ml of FITC-labeled CPE in 0.1M sodium bicarbonate. FITC-CPE was pre-mixed with mouse milk obtained from antigen-exposed (immune, PC+PG+LC group) or non-exposed (naïve) mice. After ten minutes, gavage mice were sacrificed and Peyer’s patches (PP) were harvested. PP tissues were processed for immunofluorescence as previously described. [24]

### Immunofluorescence

PP sections were stained as previously described. [24] The anti-mouse antibody CD11c-PE from eBioscience (San Jose, CA) was used for immunostaining dendritic cells in PP tissues. All incubations were done at 37°C for 30 min. Slides were mounted with ProLong Gold antifade reagent (Invitrogen, Grand Island, NY) and images were collected using a Leica SP5 ABOS scanning laser confocal microscope (Leica, Wetzlar, Germany). Images were processed using the Fiji software (http://fiji.sc/). [25]

### Statistical analysis

We performed a one-way ANOVA followed by a Newman Keuls test for all pairwise comparisons if the data were normally distributed. For antibody dilution curves, data were analyzed by a two-way ANOVA with a Bonferroni correction for multiple comparisons. Differences between groups were analyzed by a nonparametric test followed by all pairwise comparisons if the data was not normally distributed, with a Dunn’s test for multiple comparisons. Pre-post values were analyzed by Wilcoxon signed rank test. P values <0.05, based on two-tailed tests, are considered statistical significant. All statistical analyses were performed with Prism 5.0 (GraphPad, Inc.).

## Results

### Maternal peanut exposure prior to conception results in antibody production in serum and breast milk and transfer of immunoglobulins to offspring

**Fig 2:**
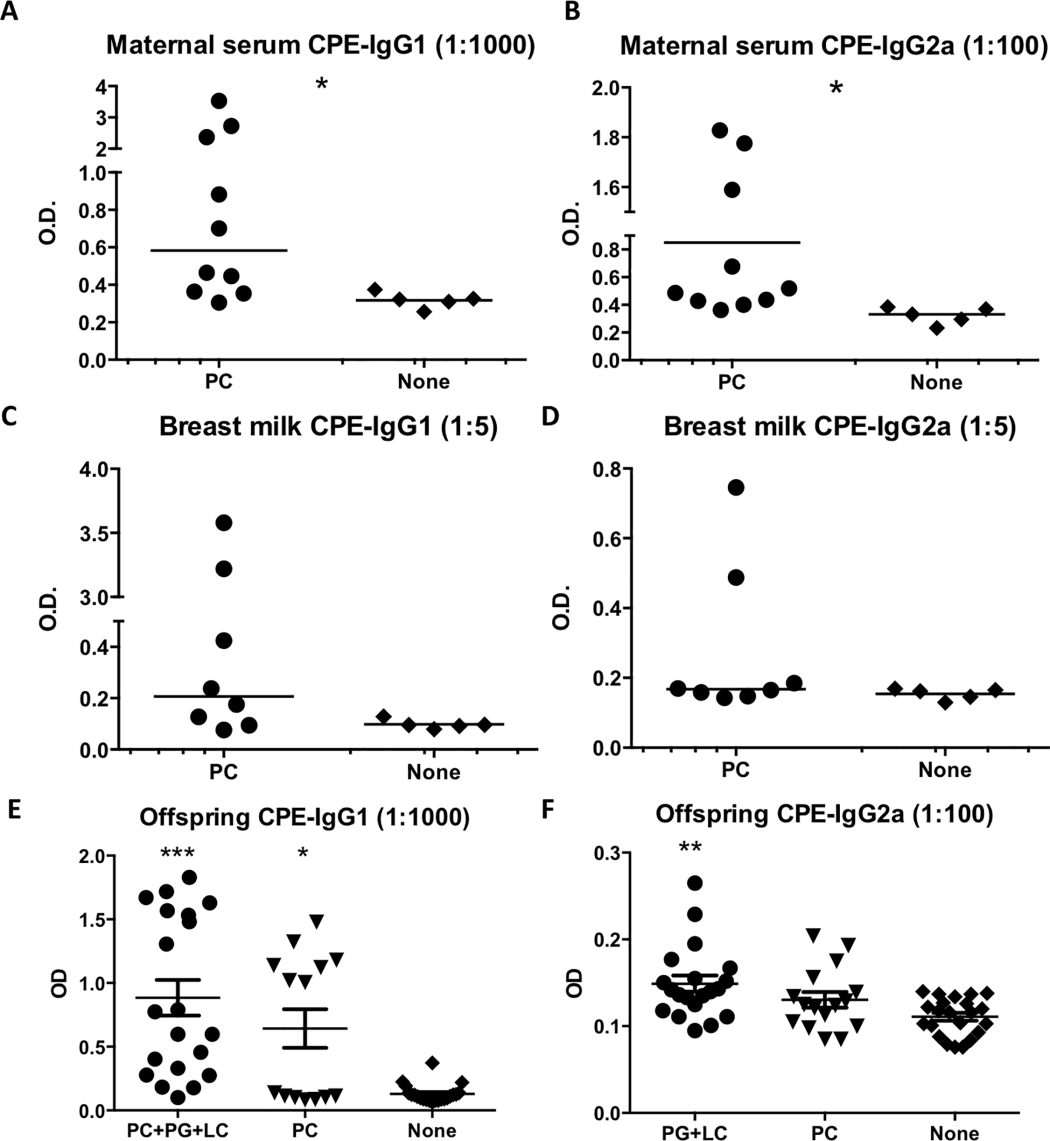
Mothers’ preconception serum (A-B) and breast milk (C-D) antibody levels in mothers who were fed peanut preconceptionally (PC) and in those who were not (None). Presensitization antibody levels at 5 weeks of age are shown (E-F) for offspring born to peanut-exposed mothers who either continued to feed peanut throughout pregnancy and lactation (PG+LC) and in those who did not (PC). The dilution curves are not shown due to the small amount of serum available. Specific IgE was undetectable (data not shown). Titles indicate sample dilution. Shown is a mean (with SEM). 5–10 mothers per group and all their offspring (>15/group) were used for each experiment. *, p<0.05; **, p<0.01; ***, p<0.001 compared to None.

To assess the efficacy of antibody production induced by oral exposure to peanut, we measured the antibody levels in maternal serum and breast milk. Three weeks after oral gavage with peanut, more than one-half of the mothers exposed to peanut prior to conception, had detectable levels of CPE-specific serum and breast milk IgG1 and IgG2a, whereas mice that were not exposed to peanut did not (**Fig 2A–2D**). Specific IgE was undetectable and these mothers were not reactive to peanut upon feeding. Also, feeding peanut throughout lactation did not further boost antibody levels (data not shown).

Maternal CPE-specific serum antibody levels were efficiently transferred to the offspring, as evidenced by elevated specific IgG1 and IgG2a levels in pups born to peanut-exposed mothers (**Fig 2E and 2F**). Pups born to non-exposed mothers had no detectable CPE-specific antibody at weaning. Maternal antibody levels positively correlated with IgG1 and IgG2a antibody levels in offspring at weaning (p<0.001 and p = 0.002, respectively, **S2 Fig**). Feeding peanut throughout lactation did not further boost antibody levels in offspring (data not shown).

### Maternal peanut exposure during pregnancy and lactation had no impact on peanut sensitization

**Fig 3:**
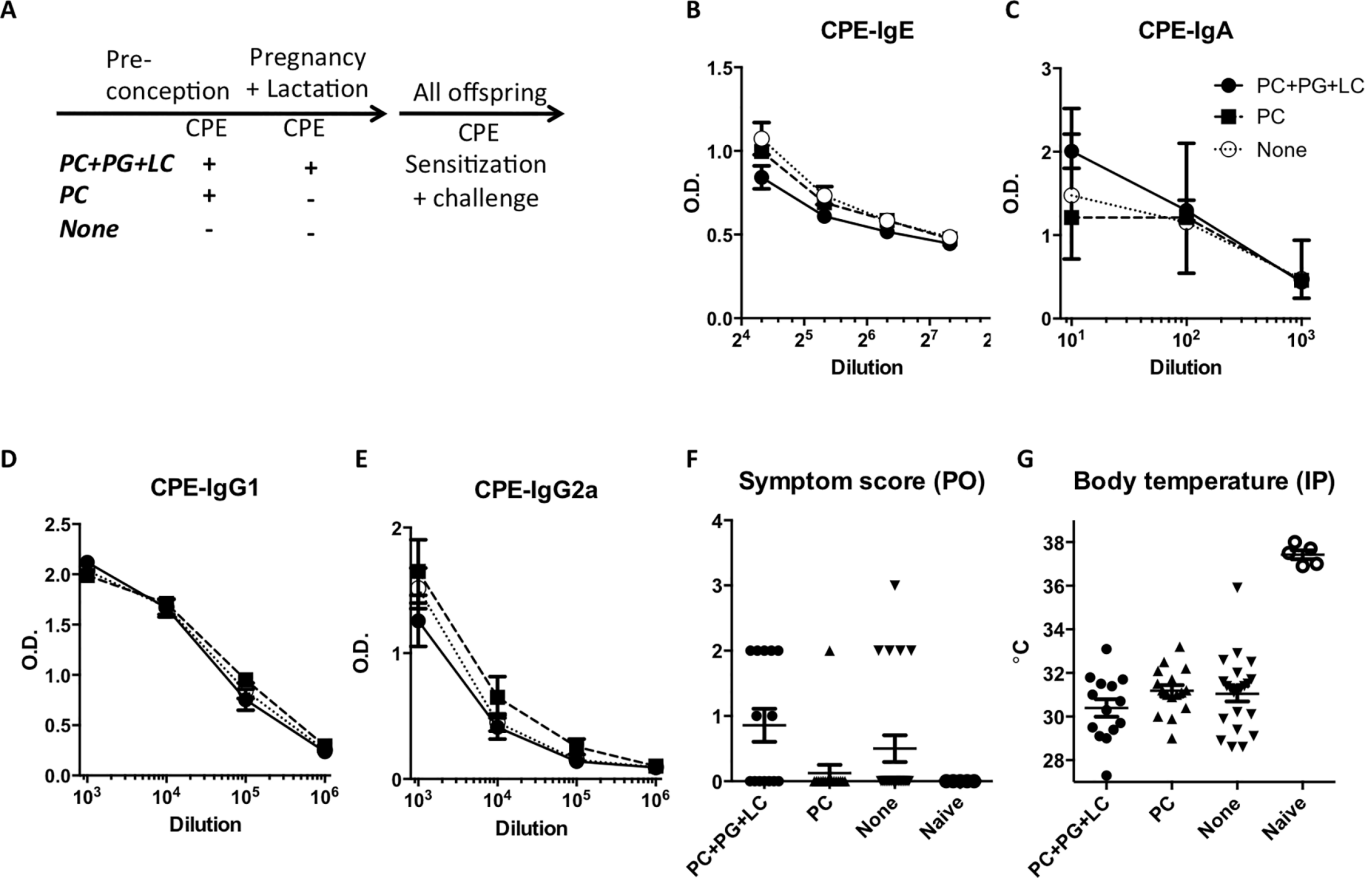
Offspring’s response to peanut sensitization based on maternal feeding of peanut. (A) Experimental protocol. Serum antibodies (B-E) and an anaphylactic response to the peanut challenge as indicated by the elevated score during the oral (PO) challenge (F) (baseline symptom score 0 in all the mice), the lowered body temperature during the intraperitoneal (IP) challenge (G), and after the peanut sensitization were assessed in offspring born to mothers exposed to peanut preconceptionally, who either continued to feed peanut during pregnancy and lactation (PC+PG+LC) and those who did not (PC). Offspring born to mothers never exposed to peanut served as controls (None). Naïve, non-sensitized mice served as controls. Shown is a mean with SEM. >15 mice were used per group.

We then assessed whether maternal feeding of peanut during pregnancy and lactation had an impact on development of peanut sensitization in the offspring. Peanut sensitization was associated with increased serum peanut-specific IgE, IgG1, IgG2a, and IgA levels, which were comparable among the 3 groups of offspring: those born to non-exposed mothers versus those born to mothers exposed to peanut prior to conception or those exposed prior to conception and throughout pregnancy and lactation (**Fig 3B–3E**). After the oral peanut challenge, the symptom scores were comparable among these 3 groups (**Fig 3F**), and similar drops in body temperature were observed after i.p. peanut challenge (**Fig 3G**). MMCP-1 and cytokine levels from splenocyte cultures were likewise comparable between the 3 groups (See **S3 Fig**). Serum levels of total IgE, specific IgE to Ara h2, and ratios of specific IgE and IgG1 to IgG2a were comparable between the three groups of mice (data not shown). Additional groups of offspring born to mothers exposed to peanut prior to conception, and either exposed to peanut only during pregnancy or only during lactation, had comparable levels of antibodies and challenge responses (data not shown). Experiments were repeated using the milk allergen BLG as an antigen. There were again no significant differences among the groups (**S4 Fig**).

### Oral tolerance to peanut develops independently of maternal peanut exposure

**Fig 4:**
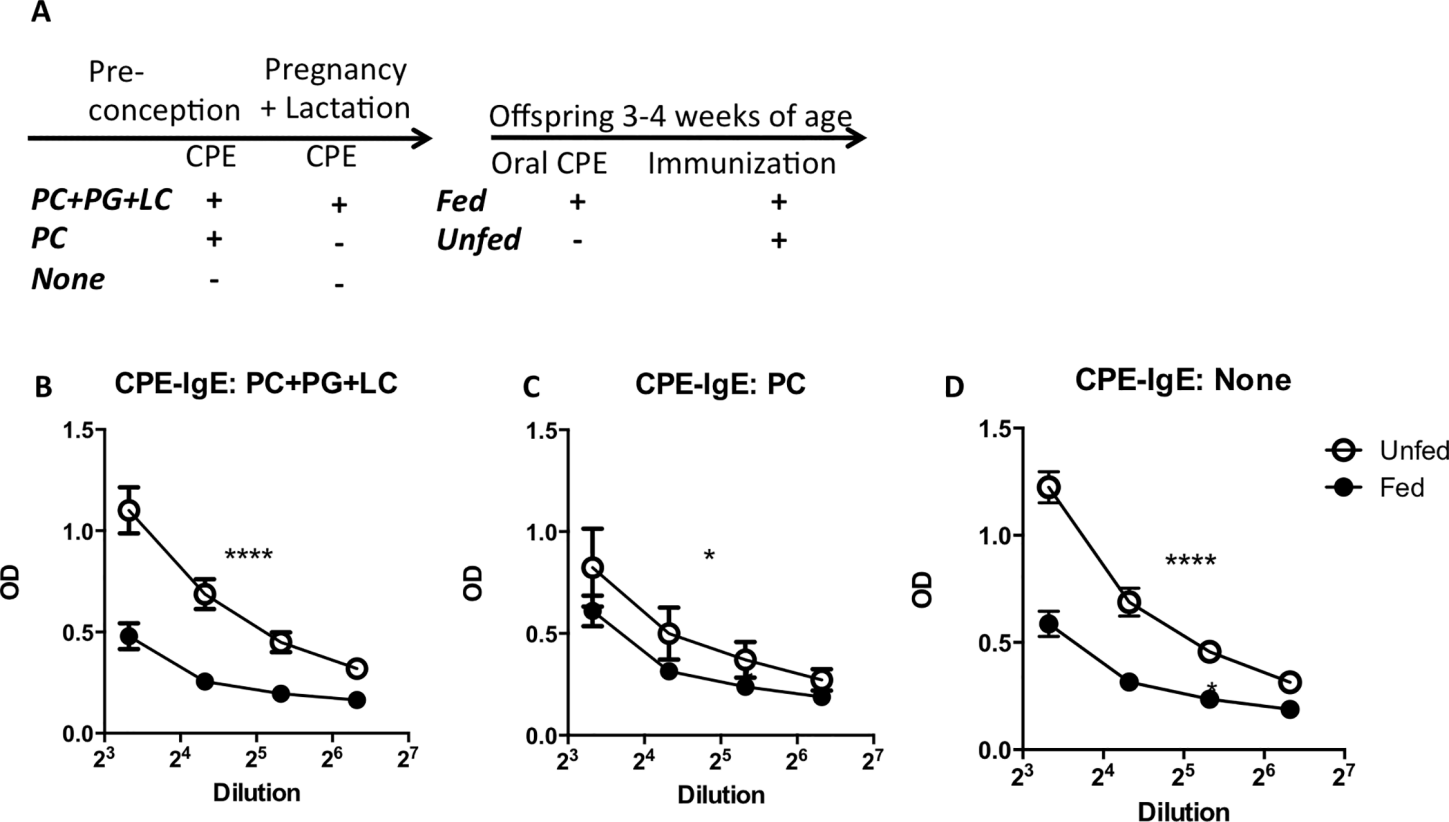
Maternal feeding of peanut does not interfere with the induction of oral tolerance in offspring. (A) Experimental protocol. (B-D) Serum IgE antibodies after peanut immunization were assessed in offspring who were either fed crude peanut extract (CPE) orally prior to immunization with CPE (Fed) or not fed (Unfed). Offspring were either born to mothers exposed to peanut preconceptionally who either continued to feed peanut during pregnancy and lactation (PC+PG+LC) or did not (PC). Offspring born to mothers that were never exposed to peanut served as controls (None). In (C), although antibody levels are similar, the dilution curves are significantly different. Shown is a mean with SEM. Each group has 10–18 mice *, p<0.05, ****, p<0.0001.

Food allergy is thought to be a combined result of failed oral tolerance and an activation of pathways that promote sensitization. We then assessed whether maternal feeding of peanut would impact offsprings’ development of oral tolerance. This was done utilizing a classic oral tolerance protocol, in which daily antigen feeding for 5 days induces a suppressed antibody response to immunization with the same antigen. We found that tolerance was induced in young mice by feeding a moderate dose (1 mg) of CPE prior to immunization, as evidenced by lower levels of specific IgE antibodies (**Fig 4**), IgG1, and IgG2a antibodies were also decreased, but not statistically different between the groups (Please see **S5 Fig**). We also showed that tolerance development was as effective in mice born to mothers exposed to peanut prior to conception and throughout pregnancy as mice born to non-peanut-exposed mothers (**Fig 4**).

### Peanut uptake into Peyer’s patch associated dendritic cells

**Fig 5:**
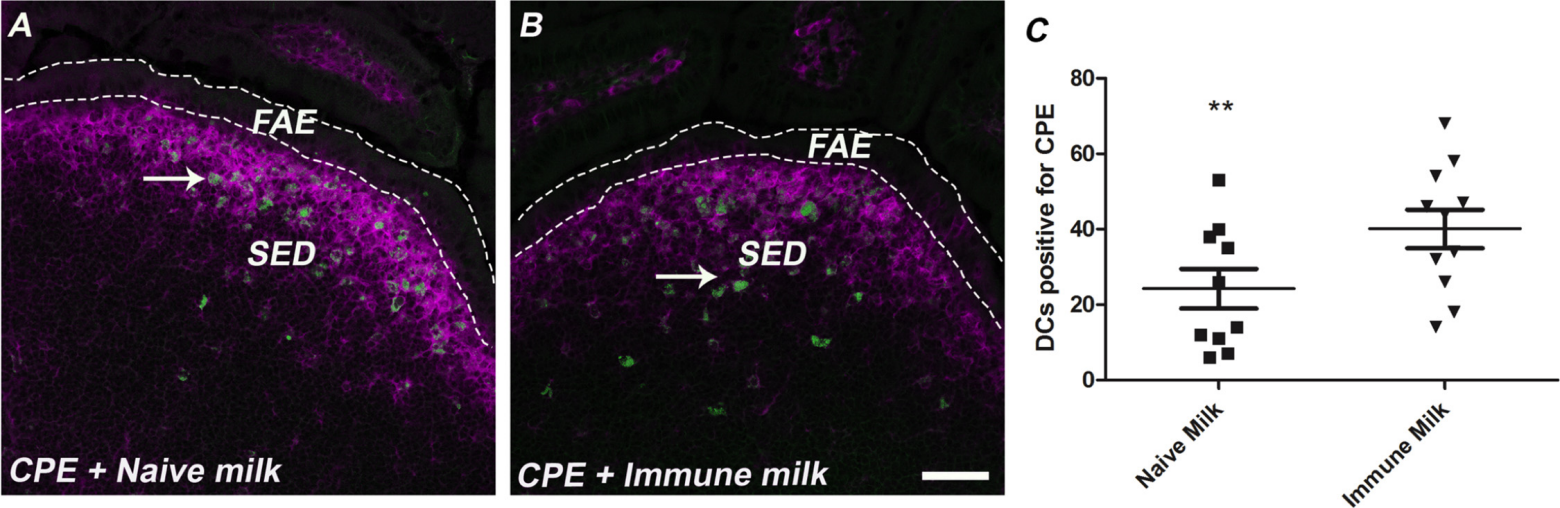
Intestinal peanut uptake to the Peyer’s patches. Mice were gavage-fed FITC-labeled CPE in the presence of naïve murine milk or immune milk. Peyer’s patches were collected 10 min later. A-B show representative sections of immunofluorescence. FAE, follicle associated epithelium; SED, subepithelial dome. FITC, CPE; Magenta, CD11c+. C) Number of DCs staining positive for CPE per patch. **, p<0.01 when compared to immune milk.

It has been reported that breast milk OVA-IgG immune complexes facilitate oral tolerance in neonates due to enhanced uptake across the mucosal barrier through the neonatal Fc receptor. [17] To examine the role of antibodies in uptake of peanut antigens following oral delivery, FITC-CPE was administered orally to 5 week-old mice in the presence of immune or naïve milk. We excised small intestinal tissues containing at least one Peyer’s patch and subjected the tissues to cryosectioning and confocal microscopy. In the context of naïve milk, we observed the accumulation of FITC-CPE within a subset of Peyer’s patch subepithelial dome dendritic cells (DCs), as evidenced by co-localization of CPE with CD11c^+^ cells (**Fig 5**). CPE was not visibly detected in the lamina propria. Co-administration of FITC-CPE with immune milk slightly enhanced FITC-CPE uptake in Peyer’s patch tissues when compared to administration of CPE with naïve milk, thus suggesting that the presence of antigen-specific IgG or IgA antibodies may facilitate transport of CPE across the follicle-associated epithelium and/or uptake by DCs.

### Oral tolerance can be induced in early life, and is neither enhanced nor impaired by concurrent exposure to immune breast milk

**Fig 6:**
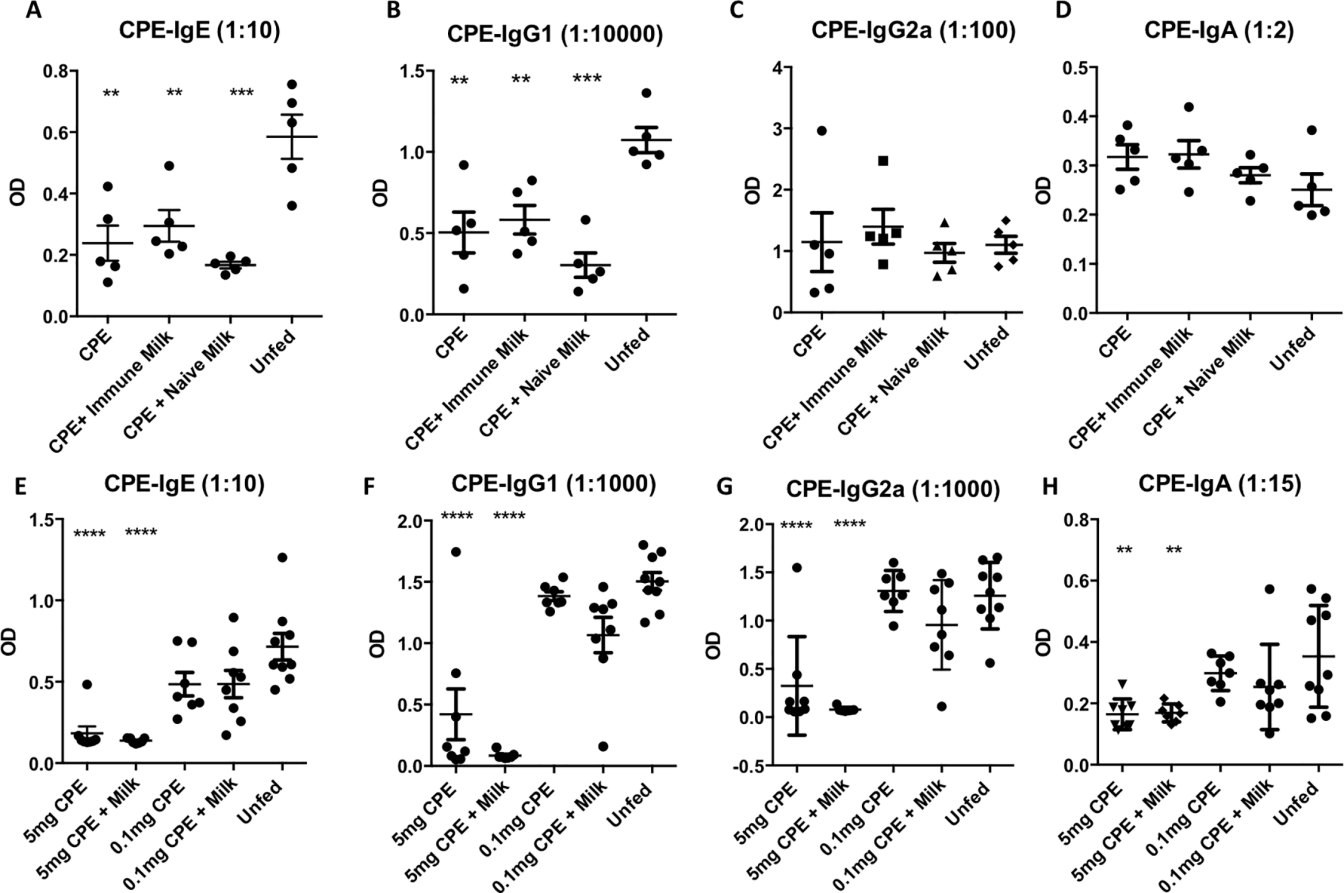
Dose-dependent induction of oral tolerance to peanut in mice prior to or after weaning. Mice post-weaning at 3–4 weeks of age (n = 5/group) (A-D), or pre-weaning at 11–12 days of age (n = 7-9/group (E-H) were fed crude peanut extract (CPE, 1 mg or as otherwise indicated) +/- murine milk (immune, unless otherwise indicated being naive) for 5 days followed by immunization with CPE. Mice immunized without prior feeding of ground peanut (Unfed) were used as untolerized controls. Shown is a mean with SEM. **, p<0.01, ***, p<0.001, ****, p<0.00001 when compared to unfed.

Lastly, because we detected enhanced peanut uptake in Peyer’s patches in the presence of peanut antibodies in breast milk, we hypothesized that direct administration of antigen to the offspring to induce oral tolerance could be influenced by concurrent immune milk exposure. We fed offspring CPE plus murine milk, either at weaning (**Fig 6A–6D**) or prior to weaning, at 11 days of age (**Fig 6E–6H**). We showed that a relatively small dose of fed CPE (1 mg) administered alone, post-weaning, was able to tolerize offspring, but co-administration with immune or naïve murine milk did not have any impact on this tolerance development (**Fig 6A and 6B**). Because the mice above were tolerized post-weaning, we then assessed whether the pre-weaning period would be more susceptible for tolerance induction by immune milk. This was done by utilizing mice at 11–12 days of life and treating them with two doses of antigen. Again, we showed that that immune milk did not significantly impact tolerance induction (**Fig 6E–6H**). We also showed that a large dose of CPE was better at inducing tolerance than small or trace quantities of antigen (**Fig 6E–6H**).

**Fig 7:**
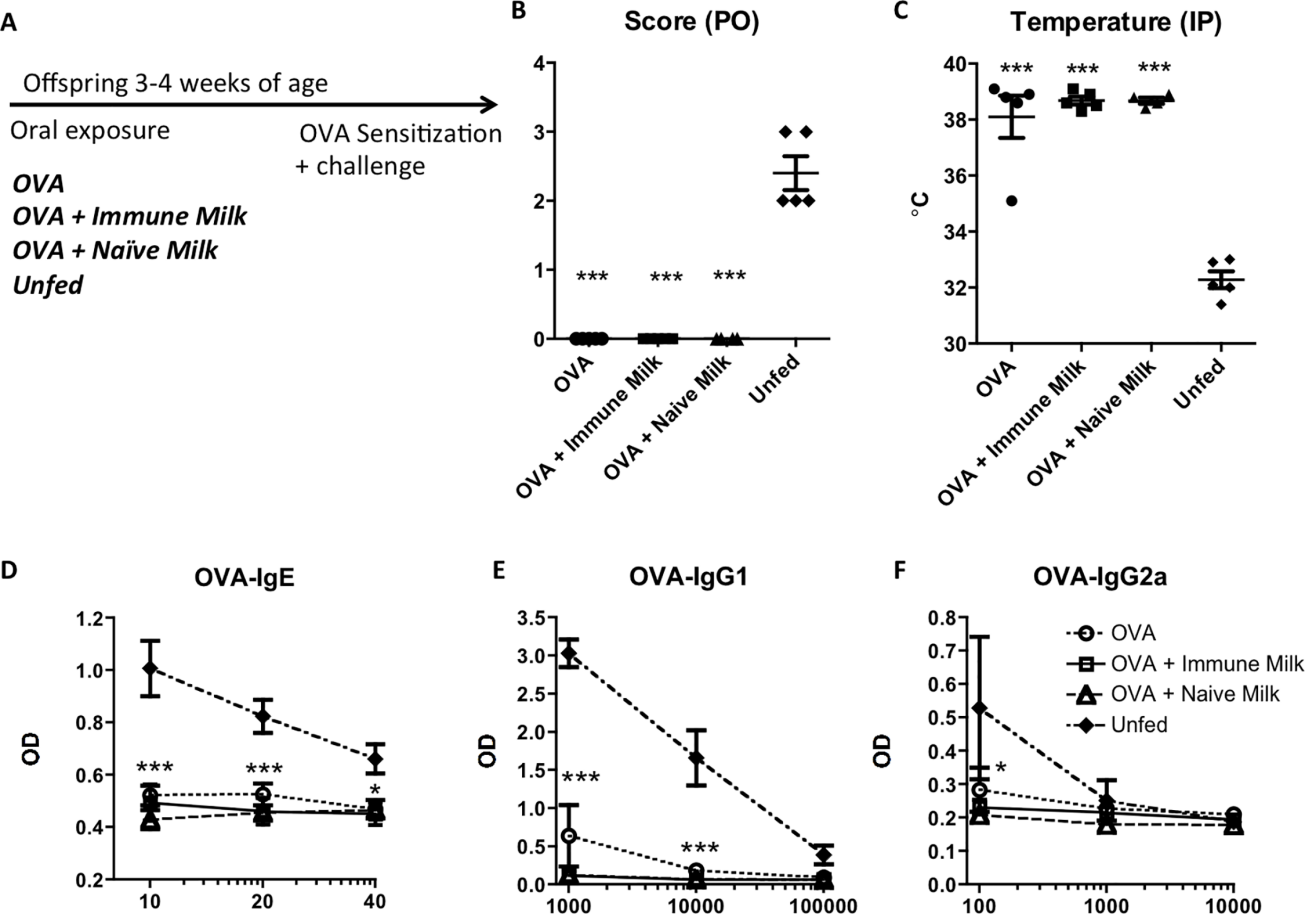
OVA epicutaneous sensitization after feeding OVA with or without murine milk. (A) Experimental protocol. Offspring come from naïve mothers. Offspring’s response to OVA challenge (B-C) and antibody responses to OVA sensitization (D-F). Mice post-weaning at 3–4 weeks of age were fed OVA +/- OVA-immune or naïve milk for 5 days followed by cutaneous sensitization with OVA. Mice immunized without prior OVA feeding (Unfed) were used as controls. X axis for D-F shows the dilution factor for the serum. Each group has 5 mice. Shown is a mean with SEM. **, p<0.01, ***, p<0.001, ****, p<0.00001 when compared to unfed.

We also assessed the impact of fed OVA co-administered with murine milk followed by OVA sensitization, with similar results (**Fig 7**).

## Discussion

The prevalence of peanut allergy has increased in children. [1–4] Peanut is commonly known as a cause for fatal and near-fatal anaphylactic reactions [26–27] and rarely outgrown. Because prevention of food allergies by maternal and infant feeding practices could serve as a simple, inexpensive approach to address the growing number of subjects with food allergies, we assessed whether maternal peanut exposure has an impact on peanut sensitization in the offspring. We report that despite vertical transfer of maternal antibodies, maternal peanut exposure and immune status had no significant impact on development of sensitization or tolerance in the offspring. In addition, we show that early introduction of peanut directly to the offspring induces dose-dependent tolerance, which is not further facilitated by co-administration of immune breast milk. These findings suggest that (i) the maternal diet plays little role in the development of peanut allergy and (ii) tolerance can be induced early, even pre-weaning, by introducing moderate amounts of peanut directly to the infant, with or without concurrent exposure to immune breast milk. Our study does not address the question of whether breastfeeding overall has an impact on sensitization or tolerance, because all the offspring were breastfed.

Our results differ from several previous animal studies that have shown protection with maternal exposure against sensitization to inhaled or ingested antigens. They have mostly been performed using OVA as an antigen in asthma models [15–18], as opposed to an oral peanut allergy model. Peanut, as opposed to OVA, activates innate immunity and complement [28–29], which may be related to greater clinically evident allergenicity. However, our studies with two other common food allergens, cow’s milk β-lactoglobulin and hen’s egg OVA, support our findings. Only one study to date has assessed maternal peanut exposure and using mothers sensitized to peanut showed protection provided by low dose, perinatal maternal peanut exposure against peanut anaphylaxis during first exposure. Protection was associated with increased peanut-specific IgG2a in the offspring, [14] related to co-administration of peanut with a mucosal adjuvant. In the current study, peanut was fed to mothers perinatally without a mucosal adjuvant, which is more relevant to the human population in which peanut ingestion typically occurs as part of a diet without co-administration of an adjuvant. Also, mothers who were allergic to peanut would not be ingesting peanut. Consistently, no enhanced IgG2a responses were detected in the current study, indicating that adjuvant use may explain the different outcomes between the studies. Different outcome may also be due to the fact that whereas the current model was IgE-mediated, anaphylaxis during first exposure in the previous model was IgG_1_-mediated. Also, because patients with peanut allergy strictly avoid the allergen, our study determined that the impact of maternal ingestion of peanut in mothers with previous exposure and presence of specific antibodies to peanut are associated with maternal tolerance, not sensitization.

In humans, there is likewise great controversy regarding the impact of maternal avoidance diets on allergy prevention. [30–33] Unlike the previous recommendations, [34] the revised maternal and infant feeding guidelines avoid strong recommendations on whether foods of high allergenic potential should be ingested during lactation. [35] More recently, emerging observational studies have created even more controversy. [11–13] Recently, two large US cohorts performed in general populations [12–13] reported that a maternal diet including peanut and tree nuts during or around pregnancy could be associated with a reduced risk to peanut or tree nut allergy in offspring. However, when nut allergic mothers were assessed separately, one study reported a trend for an association between maternal consumption of tolerated nuts and increased risk of peanut or tree nut allergy in offspring. [13] This is consistent with findings in a human cohort of high-risk infants with possible or likely cow’s milk or egg allergy that reported maternal peanut ingestion during pregnancy associated with increased peanut sensitization. [11] Because controlled clinical trials are lacking, confounding factors such as the infant’s diet and other environmental exposures may partly explain the differences in findings between cohorts.

The capacity of food antigens in breast milk to sensitize or tolerize is largely unknown. Peanut proteins [7–8] and several other dietary proteins, including cow’s milk, hen’s egg and wheat allergens, have been detected in human and murine milk. [7, 8, 15] We anticipated that peanut would also be transmitted in murine milk, although in small quantities. These levels did not seem to play a major role in sensitization or tolerance induction. Due to the fact that levels of dietary antigens present in breast milk are generally very low with many mothers having undetectable levels, [7, 8, 10] we also assessed the potential of larger doses of peanut co-administered with immune milk in inducing tolerance. We showed that large doses of peanut were better at inducing tolerance to peanut in young mice than small doses or trace amounts and that tolerance induction was not impacted by co-administration with immune milk. This suggests that low levels of dietary antigens present in breast milk may not be sufficient to induce tolerance and that doses larger than trace amounts are required. This was surprising in light of the finding that milk antibodies enhanced peanut uptake in Peyer’s patches. While animal studies are not a surrogate for human trials, our results suggest that the impact of maternal dietary antigens may be small compared to other factors, including epicutaneous exposure, which has been associated with sensitization [21, 36–38] and early oral exposure, as recently shown in the LEAP study to lead to tolerance induction. [13] Our findings further support the early introduction of foods directly to infants for development of tolerance,and although the exact “window of opportunity” cannot be extrapolated from mouse studies, introduction may be beneficial even pre-weaning without a risk of sensitization.

Our finding that breast milk does not appear to facilitate tolerance development in offspring pre- or post-weaning is in disagreement with a recent study, which showed that low quantities of immunologically active peanut allergens were secreted in human breast milk and co-administration of peanut with breast milk was associated with partial tolerance to peanut when administered to young mice prior to weaning. [8] The authors suggested that protection may be transferred via human milk antibodies and a neonatal γ receptor (FcRn), which may bind antibodies from different species. The main difference, compared to our study, was that we administered breast milk from the same species, namely murine milk, to assure that receptors facilitating antibody uptake in the neonatal gut would be appropriate to the species used.

During maternal exposure, handling pregnant mothers and gavaging these mice consistently can induce stress related changes in immune responses in the pups. A limitation to this study is that the control group was not gavaged a vehicle as compared to the pregnant mothers who were gavage fed peanut three times a week. However, mothers in each group were handled frequently and additionally stressed on a weekly or twice-a-week basis, due to milking and blood draws. Furthermore, because sensitization may be dependent on presensitization IgG levels, and offsprings’ presensitization IgG levels were highly variable, the conclusions might have been different if only offspring with high IgG1 levels would have been taken into account. Due to initial study design not accounting for this, the experiments were not sufficiently powered to perform such analyses.

In summary, despite vertical transfer of maternal IgG antibodies, we found that ante- and postnatal maternal peanut exposure does not impact peanut sensitization or oral tolerance induction in offspring, which suggests that no dietary restrictions are needed for pregnant and breastfeeding mothers in order to prevent food allergy. However, there is emerging evidence to suggest that early peanut introduction directly to infants is beneficial in prevention of peanut allergy, providing rationale for current interim feeding guidelines. [39] Furthermore, large doses of peanut were better at inducing tolerance to peanut than small doses and tolerance induction was not impacted by co-administration of immune milk either pre- or post-weaning, suggesting that trace amounts of dietary protein detected in breast milk may not be sufficient to induce significant tolerance. Randomized controlled clinical studies are needed to address the impact of maternal exposure to peanut and the “window of opportunity” for early introduction of foods on allergy risk in humans.

## Supporting Information

S1 Fig Detailed experimental protocol. (A) To assess the impact of the maternal diet, mothers were pre-conceptionally exposed to crude peanut extract (CPE) (Immune Mothers). During pregnancy and lactation, they were divided into those who continued to feed CPE (PG+LC) and those who did not (None). Non-CPE-exposed mothers that did not feed CPE pre- or post-conception served as controls. After weaning, offsprings’ responses to peanut sensitization (B) or oral tolerance induction (C) were assessed. (D) To assess the role of murine milk, oral tolerance induction or sensitization to CPE was assessed in young mice pre- or postweaning by feeding CPE alone or with murine milk from immunized mothers.
(PDF)

S2 Fig Offsprings’ presensitization antibodies. Antibodies were assessed after weaning and before immunization in offspring born to immune mothers who fed peanut during pregnancy and in those who did not (A-B). Offspring antibodies are plotted according to maternal antibodies, being either high or low. Specific IgE or IgA were undetectable. *, p<0.05, **,p<0.01, ***, p<0.001.
(PDF)

S3 Fig Offspring’s response to peanut sensitization based on maternal feeding of peanut. (A) Serum MMCP levels were measured 30 min after intraperitoneal peanut challenge. (B-D) Cytokine levels were measured in splenocyte cultures stimulated with peanut for 72 hours. In offspring born to mothers exposed to peanut preconceptionally, who either continued to feed peanut during pregnancy and lactation (PC+PG+LC) and in those who did not (PC). Offspring born to mothers never exposed to peanut served as controls (None). Naïve, non-sensitized mice served as controls. Shown is a mean with SEM.
(PDF)

S4 Fig Offspring’s response to BLG sensitization based on maternal feeding of BLG. Experimental protocol (A). Mothers’ preconception serum (B) and breast milk (C) antibody levels in mothers who were fed BLG preconceptionally (BLG Fed) and in those who were not (Naive). Offspring were sensitized to BLG and serum antibodies (D-F) and symptom scores were measured after oral (PO) feeding (G) and body temperature measured after intraperitoneal (IP) injection of BLG (H), in offspring born to mothers exposed to BLG only preconceptually (PC) and in those who continued to feed BLG during pregnancy and lactation (PC+PG+LC). Offspring born to mothers never exposed to BLG served as controls (None). Shown is a mean with SEM. Each group has 4–12 mice. **, p<0.01.
(PDF)

S5 Fig Maternal feeding of peanut does not interfere with the induction of oral tolerance in offspring. (A-C) Serum CPE-specific IgG1 and (D-F) IgG2a antibodies after PN immunization were assessed in offspring who were either fed PN orally prior to immunization (Fed) or not fed (Unfed). Offspring were either born to mothers exposed to CPE preconceptionally who either continued to feed peanut during pregnancy and lactation (PC+PG+LC) and in those who did not (PC). Offspring born to mothers never exposed to peanut served as controls (None). Shown is a mean with SEM. Each group has 10–18 mice. *, p<0.01, ***, p<0.001.
(PDF)
