# Supplementary material for: Role of Maternal Dietary Peanut Exposure in Development of Food Allergy and Oral Tolerance
Source: PLoS One. 2015 Dec 10;10(12):e0143855. doi: 10.1371/journal.pone.0143855 (PMC4675539; doi:10.1371/journal.pone.0143855)
Supplement: S1 Fig — (A) To assess the impact of the maternal diet, mothers were pre-conceptionally exposed to crude peanut extract (CPE) (Immune Mothers). During pregnancy and lactation, they were divided into those who continued to feed CPE (PG+LC) and those who did not (None). Non-CPE-exposed mothers that did not feed CPE pre- or post-conception served as controls. After weaning, offsprings’ responses to peanut sensitization (B) or oral tolerance induction (C) were assessed. (D) To assess the role of murine milk, oral tolerance induction or sensitization to CPE was assessed in young mice pre- or postweaning by feeding CPE alone or with murine milk from immunized mothers. (PDF) [file pone.0143855.s001.pdf]

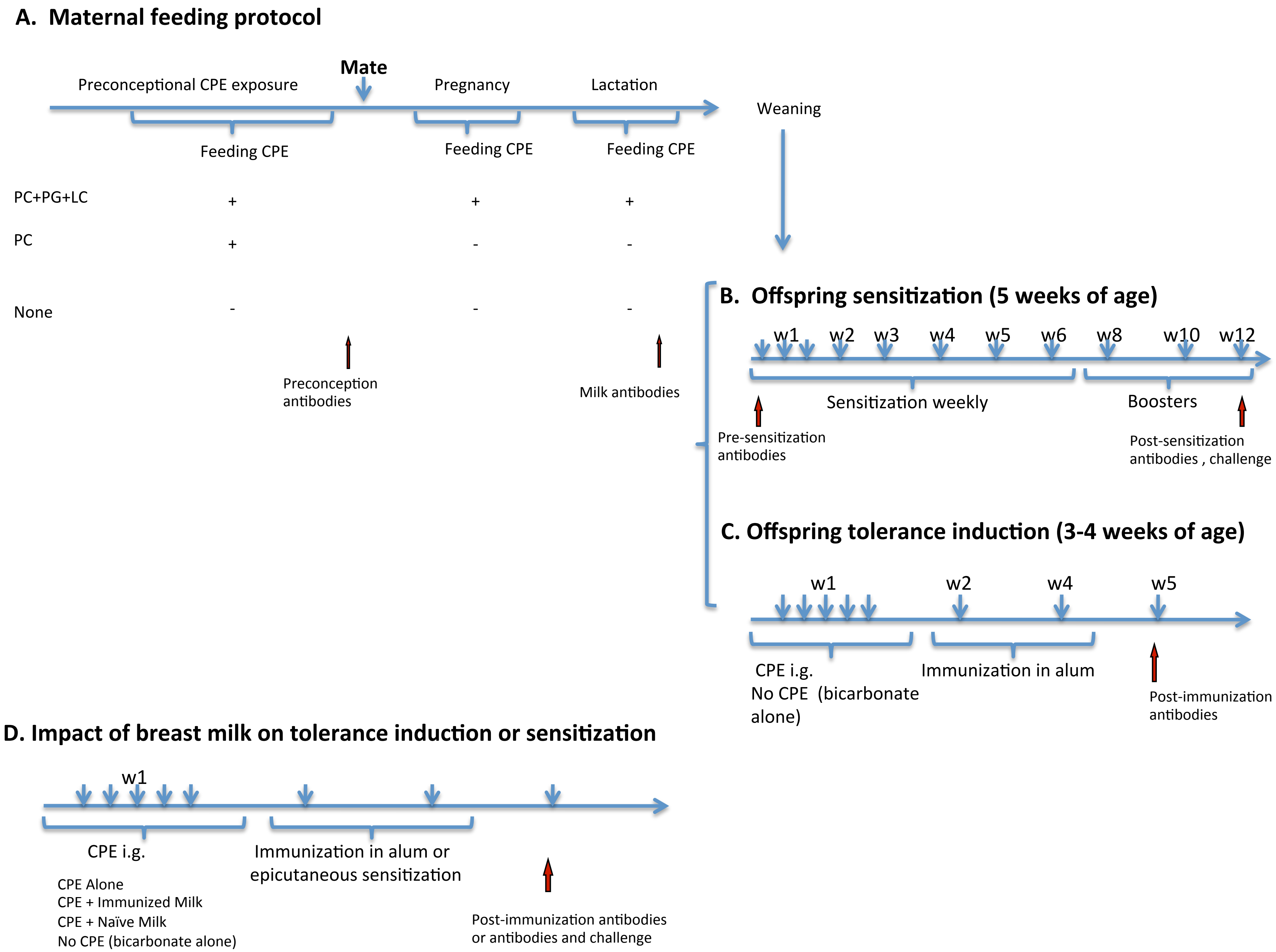
A. Maternal feeding protocol
Mate
Preconceptional CPE exposure
Pregnancy
Lactation
Weaning
Feeding CPE
Feeding CPE
Feeding CPE
PC+PG+LC + + +
PC + - -
None - - -
Preconception antibodies
Milk antibodies
B. Offspring sensitization (5 weeks of age)
w1 w2 w3 w4 w5 w6 w8 w10 w12
Sensitization weekly
Boosters
Pre-sensitization antibodies
Post-sensitization antibodies , challenge
C. Offspring tolerance induction (3-4 weeks of age)
w1 w2 w4 w5
CPE i.g.
No CPE (bicarbonate alone)
Immunization in alum
Post-immunization antibodies
D. Impact of breast milk on tolerance induction or sensitization
w1
CPE i.g.
CPE Alone
CPE + Immunized Milk
CPE + Naïve Milk
No CPE (bicarbonate alone)
Immunization in alum or epicutaneous sensitization
Post-immunization antibodies or antibodies and challenge
