# Supplementary figures and images for: Role of Maternal Dietary Peanut Exposure in Development of Food Allergy and Oral Tolerance
Source: PLoS One. 2015 Dec 10;10(12):e0143855. doi: 10.1371/journal.pone.0143855 (PMC4675539; doi:10.1371/journal.pone.0143855)

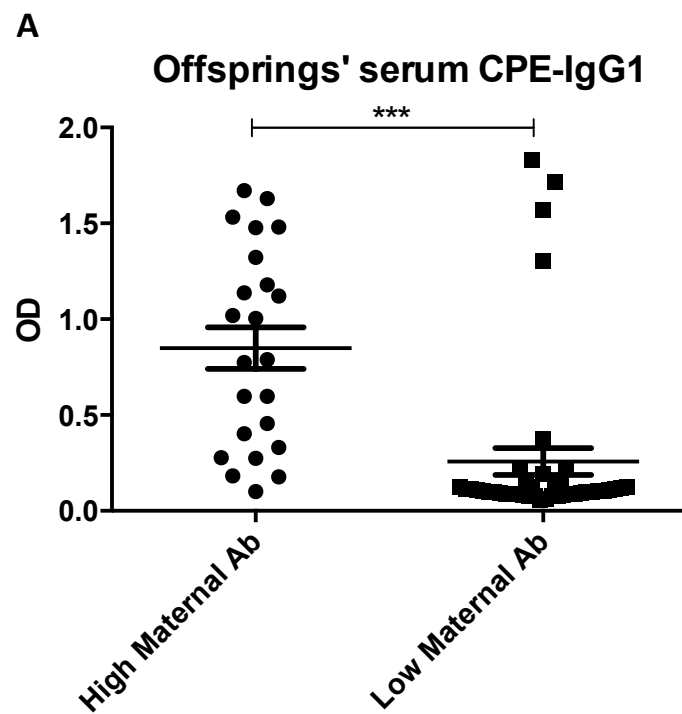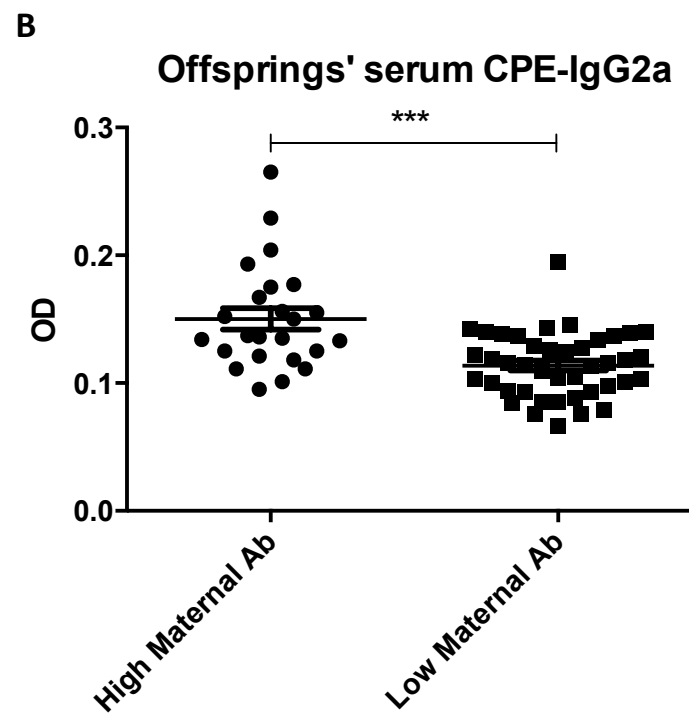

Supplement: S2 Fig — Antibodies were assessed after weaning and before immunization in offspring born to immune mothers who fed peanut during pregnancy and in those who did not (A-B). Offspring antibodies are plotted according to maternal antibodies, being either high or low. Specific IgE or IgA were undetectable. *, p<0.05, **,p<0.01, ***, p<0.001. (PDF) [file pone.0143855.s002.pdf]

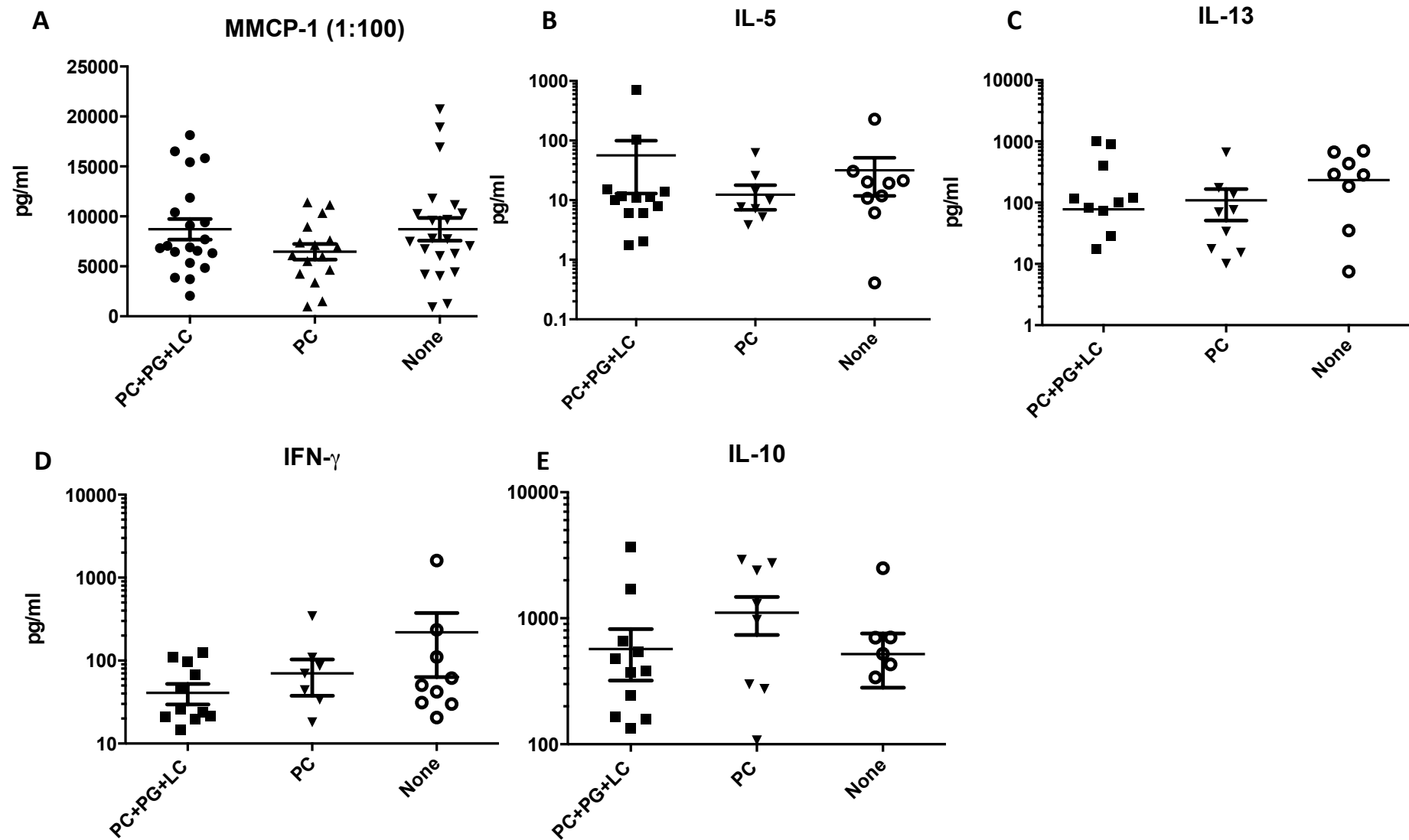

Supplement: S3 Fig — (A) Serum MMCP levels were measured 30 min after intraperitoneal peanut challenge. (B-D) Cytokine levels were measured in splenocyte cultures stimulated with peanut for 72 hours. In offspring born to mothers exposed to peanut preconceptionally, who either continued to feed peanut during pregnancy and lactation (PC+PG+LC) and in those who did not (PC). Offspring born to mothers never exposed to peanut served as controls (None). Naïve, non-sensitized mice served as controls. Shown is a mean with SEM. (PDF) [file pone.0143855.s003.pdf]

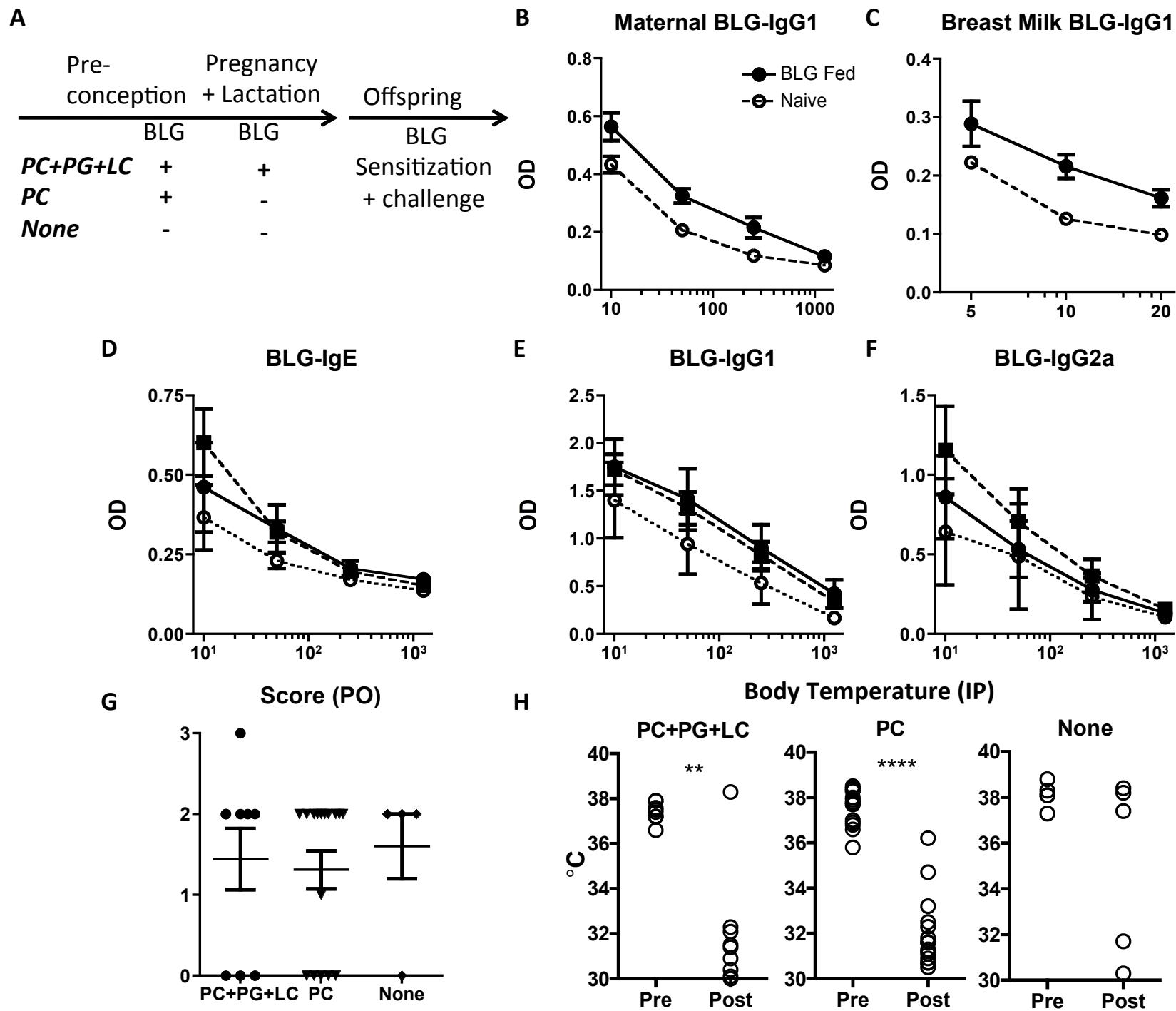

Supplement: S4 Fig — Experimental protocol (A). Mothers’ preconception serum (B) and breast milk (C) antibody levels in mothers who were fed BLG preconceptionally (BLG Fed) and in those who were not (Naive). Offspring were sensitized to BLG and serum antibodies (D-F) and symptom scores were measured after oral (PO) feeding (G) and body temperature measured after intraperitoneal (IP) injection of BLG (H), in offspring born to mothers exposed to BLG only preconceptually (PC) and in those who continued to feed BLG during pregnancy and lactation (PC+PG+LC). Offspring born to mothers never exposed to BLG served as controls (None). Shown is a mean with SEM. Each group has 4–12 mice. **, p<0.01. (PDF) [file pone.0143855.s004.pdf]

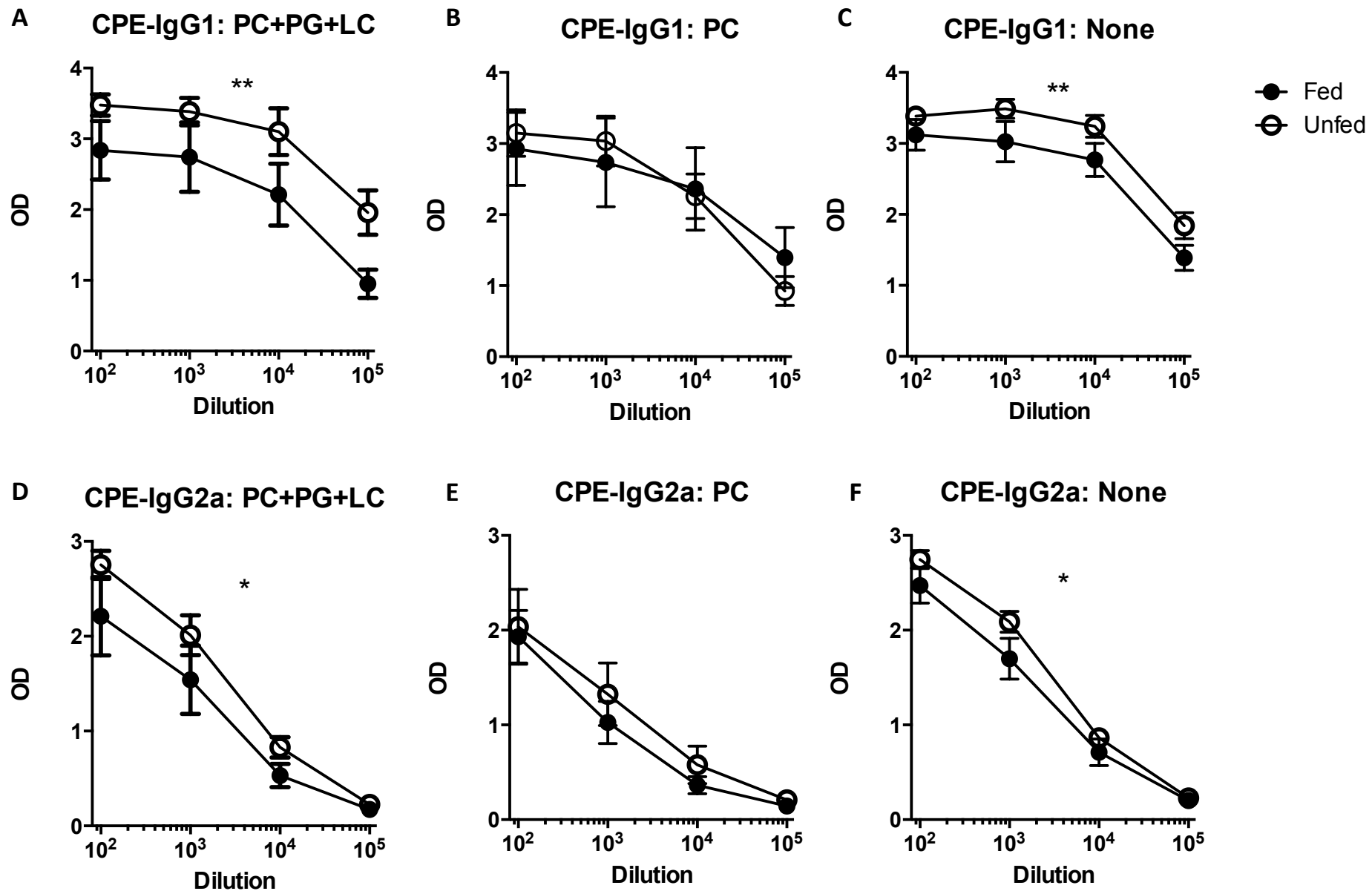

Supplement: S5 Fig — (A-C) Serum CPE-specific IgG1 and (D-F) IgG2a antibodies after PN immunization were assessed in offspring who were either fed PN orally prior to immunization (Fed) or not fed (Unfed). Offspring were either born to mothers exposed to CPE preconceptionally who either continued to feed peanut during pregnancy and lactation (PC+PG+LC) and in those who did not (PC). Offspring born to mothers never exposed to peanut served as controls (None). Shown is a mean with SEM. Each group has 10–18 mice. *, p<0.01, ***, p<0.001. (PDF) [file pone.0143855.s005.pdf]
